# Novel approach to pleurodesis with 50 % glucose for air leakage after lung resection or pneumothorax

**DOI:** 10.1007/s00595-015-1223-2

**Published:** 2015-07-24

**Authors:** Kosuke Fujino, Yamato Motooka, Takamasa Koga, Hironobu Osumi, Eri Matsubara, Hidekatsu Shibata, Koei Ikeda, Kenji Shiraishi, Takeshi Mori, Koji Hayashi, Kentaro Yoshimoto, Jyoeji Wakimoto, Ichiro Kubota, Makoto Suzuki

**Affiliations:** Department of Thoracic Surgery, Graduate School of Medical Sciences, Kumamoto University, 1-1-1 Honjo, Kumamoto, Japan; Department of Surgery, Kumamoto Red Cross Hospital, Kumamoto, Japan; Department of Thoracic Surgery, National Hospital Organization Minamikyushu Hospital, Kagoshima, Japan

**Keywords:** Pleurodesis, Lung resection, Pneumothorax, Postoperative care, 50 % Glucose solution

## Abstract

**Purpose:**

Pleurodesis is performed in patients demonstrating air leakage after lung resection and in those with pneumothorax who must avoid surgery. However, there have so far been very few reports of pleurodesis with 50 % glucose. We herein examined the feasibility and effectiveness of this novel pleurodesis technique.

**Methods:**

Thirty-five patients after lung resection and 11 pneumothorax patients without surgery were treated with pleurodesis using 50 % glucose. Approximately, 200 mL of 50 % glucose solution was injected into the pleural space and repeated until the air leakage stopped. Cases in which the air leakage did not stop after three injections were considered to be unsuccessful and subsequently treated with conventional pleurodesis using OK-432.

**Results:**

Thirty-nine patients were successfully treated with 50 % glucose, although 7 patients required further treatment with OK-432. The unsuccessful group had some pulmonary comorbidities (*P* < 0.001), and the pleural effusion volume after pleurodesis was less than that in the successful group (*P* < 0.001). Although the air leakage did not stop in unsuccessful patients, the amount of air leakage markedly decreased. A temporary elevation of the blood sugar level was observed in 20 patients, but no other side effects had appeared.

**Conclusions:**

Pleurodesis with 50 % glucose is an easy, safe, and effective treatment modality. It is therefore considered to be a useful alternative method for pleurodesis.

## Introduction

Pleurodesis is commonly performed for patients with pulmonary air leakage after lung resection and for those who should avoid surgery [1–5]. Various methods using various preparations have been reported; OK-432 is most often used for pleurodesis in Japan, and talc is used in Western countries. Although several studies have reported the efficacy and the safety of talc, its use had not been approved in Japan, as there have also been cases of talc that contained asbestos. However, only for patients with malignant pleural effusions, the use of purified talc was approved in December 2013 in Japan; however, OK-432 has been primarily used for postoperative air leakage and pneumothorax [6–9]. Pleurodesis using OK-432 is currently the most commonly performed technique at our hospital as well. These methods aim for adhesion through an inflammatory reaction caused by pleural stimulation instilled by the preparations [10, 11]. Chemical pleurodesis occasionally causes marked discomfort in patients, such as severe chest pain and a fever, and there are several reports of severe complications [12–14]. Therefore, it is necessary to develop safer agents for the treatment of pleurodesis. Therefore, we actively perform pleurodesis at our hospital using a 50 % glucose solution, which has no medical properties, has few side effects, is low cost, and is an easy procedure. Since large clinical trials have not been conducted for pleurodesis using a highly concentrated glucose solution, its effectiveness and side effects remain unclear. Although there have been several reports of the effectiveness of pleurodesis using a hypertonic glucose solution for secondary pneumothorax or chylothorax [15–17], this is the first report of the use of this procedure for all possible indications for pleurodesis, including postoperative air leakage after surgery and pneumothorax. In this study, the clinical characteristics of patients who underwent pleurodesis using 50 % glucose solution at our hospital and its effectiveness were evaluated.

## Materials and methods

The patients’ background characteristics, clinical condition, time to drain withdrawal, and side effects were examined in a total of 46 patients, 35 postoperative patients who underwent pleurodesis due to air leakage after lung resection, and 11 pneumothorax patients who underwent pleurodesis without undergoing surgery, from April 2012 to March 2014. During this period, although 53 patients had to undergo pleurodesis, 7 with diabetes mellitus were excluded, since highly concentrated glucose solution administered in the pleural space may cause an increase in the blood sugar level. Pleurodesis was indicated for the treatment of patients who were found to have air leakage with a chest tube under a water seal on the third postoperative or drainage day. In the conventional method, we conducted pleurodesis with OK-432 after the seventh postoperative day. In the present study, we performed pleurodesis with a 50 % glucose solution on the third postoperative day for the purpose of earlier removal of the chest tube. Because the intra-thoracic administration of highly concentrated glucose solution is not currently approved in Japan, patients who agreed to this method were included after obtaining their informed consent and approval from the Institutional Review Board of National Hospital Organization Minamikyushu Hospital.

### Pleurodesis

Local anesthesia of the parietal pleura was achieved by the injection of 10 mL of 1 % lidocaine into the pleural space, after which 200 mL of a 50 % glucose solution were injected. The chest tube was clamped for 2 h; however, in cases with a large air leakage, the tube was connected by a water seal and placed in a position higher than the patient’s body. During this period, the patients were asked to change their position on the bed every 15 min. Two hours after administration, the chest tube was opened and left on water seal, and the pleural effusion was thus drained. The drainage volume was measured every 2 h, and the blood sugar levels were measured after the first hour and then again 3 and 6 h later. Eating was prohibited 3 h before and after administration. Cases in which the air leakage did not stop were re-administered the 50 % glucose solution, and after three injections of the 50 % glucose solution the cases were considered to be unsuccessful and subsequently treated with conventional pleurodesis using OK-432.

### Statistical analyses

The SPSS ver18 software program (SPSS Inc, Chicago, IL, USA) was used for statistical analyses. The drainage volume data were assessed using the Mann–Whitney *U* test. Other data were evaluated by Fisher’s exact test. A *P* value less than 0.05 was considered to be statistically significant.

## Results

The characteristics and results of the 46 patients, who had a mean age of 69 years (range 17–86 years), are shown in Table 1. There were 39 patients in the successful group and 7 in the unsuccessful group. The 7 unsuccessful patients had more pulmonary comorbidities than those in the successful group (*P* < 0.001). All of the patients who did not have pulmonary comorbidities and 14 of 21 patients who had pulmonary comorbidities were successfully treated with the 50 % glucose solution. Although the air leakage did not stop in the unsuccessful patients, the amount of air leakage markedly decreased. The 7 unsuccessful patients were then treated with conventional pleurodesis using 5 KE of OK-432, and the drain was removed. The mean frequency for administering the 50 % glucose solution was 1.4 times (range 1–3 times) in the successful group (Table 2). The mean of the drainage period was 6.8 days (range 4–8 days) in the successful group and 11.3 days (range 8–14 days) in the unsuccessful group. After opening of the drain, a large amount of drainage was observed. The drainage volume was significantly greater in the successful group than in the unsuccessful group (*P* < 0.001, Table 3). The patient with the largest amount of drainage drained 780 mL in the first 2 h; however, no hemodynamic deterioration or symptoms of dehydration occurred in any patient. The blood sugar levels are shown in Table 4. The blood sugar levels of 20 patients 1 h after injection were greater than 250 mg/dL. Although the patients were permitted a meal 3 h after the injection, the blood sugar level was below 250 mg/dL in all patients after 6 h. All patients were given NSAIDs prophylactically; however, additional doses of analgesic were required in 9 patients due to chest pain after administration of the glucose solution. However, the pain was temporary and not prolonged. Meanwhile, 6 patients in the unsuccessful group had a fever of at least 38 °C, and 5 patients in this group had prolonged severe chest pain for more than 3 days after the injection of OK-432. No other side effects were observed even in 10 patients with interstitial pneumonia or 6 elderly patients more than 80 years of age.

**Table 1:** Patients’ characteristics

	Total	Successful	Unsuccessful	*P* value
Number of patients	46	39	7	
Sex				
Males	31	24	7	0.078
Females	15	15	0	
Operative diagnosis				
Lung cancer	29	24	5	1.000
Interstitial lung disease	4	3	1	0.496
Pneumothorax	13	12	1	0.654
Operation				
Lobectomy	25	21	4	1.000
Segmentectomy	1	0	1	0.152
Partial resection	9	7	2	0.609
Chest drainage	11	11	0	0.171
Comorbidity				
None	25	25	0	<0.001
Present	21	14	7	
Emphysema	11	8	3	0.33
Interstitial lung disease	5	4	1	1.000
Emphysema + interstitial lung disease	5	2	3	0.0199

**Table 2:** The frequency of administration of the 50 % glucose solution

	Successful	Unsuccessful
One time	28	0
Two times	8	0
Three times	3	7

**Table 3:** The mean drainage volume after pleurodesis

	Successful	Unsuccessful	*P* value
Two hours	372.6	175.9	0.001
Four hours	153.9	66.9	0.004
Six hours	58.5	38.8	0.572
Eight hours	20.8	15.6	0.345
Total volume (mL)	605.7	297.2	<0.001

**Table 4:** Blood sugar levels after pleurodesis

BSL (mg/dL)	One hour	Three hours	Six hours
≤180	12	36	45
181–250	14	8	1
251–350	16	2	0
≥351	4	0	0

*BSL* blood sugar level

## Discussion

There have been very few reports on pleurodesis with a 50 % glucose solution, and no large clinical study has been previously conducted. In addition, the mechanism of pleurodesis with a 50 % glucose solution has not yet been clarified. In the present study, the pleural effusion volume after pleurodesis was significantly greater in the successful group. Thus, it can be inferred that a hypertonic glucose solution causes osmotic cell injury and inflammation when pleural cells are damaged by the osmotic pressure difference, thereby inducing the precipitation of fibrin, which completes the pleural adhesion. We are currently confirming the details of this mechanism using experimental animal models.

Regarding the side effects, a fever was not detected, and chest pain was only present at the time of injection; the pain was relieved within a few hours and was not prolonged. On the other hand, in more than 70 % of patients with failure due to air leakage who required OK-432, a fever of at least 38 °C was noted, and severe chest pain persisted for more than 3 days. The stimulation of pleural cells and the inflammatory reaction of 50 % glucose as an adhesive drug may be milder compared with OK-432; however, 39 patients were successfully treated with this method. Thus, several patients with air leakage could be treated with this method before considering the use of exogenous chemicals.

As a specific side effect of using a 50 % glucose solution, the temporal elevation of the blood sugar level was observed in 20 of 46 cases; however, the levels of all patients were less than 250 mg/dL after 6 h. Because a high blood sugar level was observed after pleurodesis even in non-diabetic patients, diabetic patients should probably not undergo this procedure. A rapid induction of pleural effusion was observed, with a peak at 2–4 h after opening of the drain; therefore, we carefully monitored the circulatory dynamics of all patients. No patients developed dehydration or blood pressure fluctuations. Because the patients were free to drink while performing the procedure, the side effects of massive pleural effusion might be minimal. Regarding cost, a 50 % glucose solution is clearly reasonable compared with other drugs and easily obtainable in daily medical practice. In addition, the risk of infection is thought to be low, considering that it is a sterilized intravenous fluid preparation and hypertonic glucose solution.

In summary, we successfully treated 39 of 46 patients with air leakage by pleurodesis with a 50 % glucose solution. This method appears to be an easy, safe, effective, and low-cost treatment. A 50 % glucose solution does not include exogenous toxic chemical substances; thus, it could be considered to be an alternative method of pleurodesis before conducting conventional chemical pleurodesis.
